# Attitude and use of herbal medicines among pregnant women in Nigeria

**DOI:** 10.1186/1472-6882-9-53

**Published:** 2009-12-31

**Authors:** Titilayo O Fakeye, Rasaq Adisa, Ismail E Musa

**Affiliations:** 1 Department of Clinical Pharmacy & Pharmacy Administration, University of Ibadan, Nigeria; 2 Pharmacy Department, Staff Clinic, Central Bank of Nigeria, Ibadan, Nigeria

## Abstract

**Background:**

The use of herbal medicines among pregnant women in Nigeria has not been widely studied.

**Methods:**

Opinion of 595 pregnant women in three geopolitical zones in Nigeria on the use of herbal medicines, safety on usage, knowledge of potential effects of herbal remedies on the fetus and potential benefits or harms that may be derived from combining herbal remedies with conventional therapies were obtained using a structured questionnaire between September 2007 and March 2008. Descriptive statistics and Fisher's exact tests were used at 95% confidence level to evaluate the data obtained. Level of significance was set at p < 0.05.

**Results:**

More than two-third of respondents [67.5%] had used herbal medicines in crude forms or as pharmaceutical prepackaged dosage forms, with 74.3% preferring self-prepared formulations. Almost 30% who were using herbal medicine at the time of the study believed that the use of herbal medicines during pregnancy is safe. Respondents' reasons for taking herbal medications were varied and included reasons such as herbs having better efficacy than conventional medicines [22.4%], herbs being natural, are safer to use during pregnancy than conventional medicines [21.1%], low efficacy of conventional medicines [19.7%], easier access to herbal medicines [11.2%], traditional and cultural belief in herbal medicines to cure many illnesses [12.5%], and comparatively low cost of herbal medicines [5.9%].

Over half the respondents, 56.6% did not support combining herbal medicines with conventional drugs to forestall drug-herb interaction. About 33.4% respondents believed herbal medicines possess no adverse effects while 181 [30.4%] were of the opinion that adverse/side effects of some herbal medicines could be dangerous. Marital status, geopolitical zones, and educational qualification of respondents had statistically significant effects on respondents views on side effects of herbal medicines [p < 0.05)] while only geopolitical zones and educational qualifications seemed to have influence on respondents' opinion on the harmful effects of herbal medicines to the fetus [p < 0.05].

**Conclusion:**

The study emphasized the wide spread use of herbal medicines by pregnant women in Nigeria highlighting an urgent need for health care practitioners and other health care givers to be aware of this practice and make efforts in obtaining information about herb use during ante-natal care. This will help forestall possible interaction between herbal and conventional medicines.

## Background

Herbal medicines can be purchased in bulk in the crude form or as refined pharmaceutical dosage forms such as capsules, tablets, concentrated extracts, teas, tinctures and decoctions. The use of herbal medicines play significant roles in the management of both minor and major illnesses [1-3] and has been influenced by patients' dissatisfaction with conventional allopathic medicines in terms of effectiveness and/or safety, satisfaction with therapeutic outcome [4,5], and the perception that herbal medicines are inherently safe. Some of the more complex reasons for preference of herbal medicines are associated with cultural and personal beliefs, philosophical views on life and health [6], as well as comparison of experiences between conventional healthcare professionals and complementary medicine practitioners [7] by patients.

The use of herbal medicine has been on increase in many developing and industrialized countries [8,9]. It is known that between 65 and 80% of the world's population use herbal medicines as their primary form of health care [10,11]. Patients who are likely to be at risk from adverse effects of herbal medicines include those who are already prone to difficulties from regularly prescribed medications namely fetus, infants and older children, the elderly, as well as pregnant and lactating women [12-18]. In developing nations most especially, regulation of sales, importation and manufacturing of herbal medicines are not subject to rigorous scrutiny in terms of safety and efficacy as is the case for conventional western/allopathic medicines [1].

Few studies on the pattern of use of herbal medicines during pregnancy showed that more than 10% of pregnant women reported the use of herbal medicinal products in Finland, Australia, and United States [19-23]. To our knowledge, only one study has been carried out in Nigeria to evaluate the use of herbal medicines among pregnant women [24]. Despite the fact that knowledge of potential side effects of many herbal medicines in pregnancy is limited [25-29], and that some herbal products may be teratogenic in human and animal models [30-33], data on the extent of women's use of herbal medicines during pregnancy is scanty especially in sub-Sahara Africa, where the legislation for distribution and purchase of herbal medicines is not as stringent as it is for conventional medicines [34].

This study was aimed at determining the proportion, prevalence of use, attitude and knowledge base of pregnant women in Nigeria *vis a vis *use of herbal medicines and potential effects of herbal remedies on the fetus. Opinion of pregnant women on the potential benefits or harms of combining herbal remedies with conventional therapies were also obtained.

## Methods

Of the six geopolitical zones in Nigeria, a state each from three zones, namely the North Central, North West and South West were chosen for the study using a major maternity hospital in each state. They were Adeoyo Maternity Hospital, Ibadan from the South West; General Hospital, Minna from the North Central and Uthman Dan Fodio University Teaching Hospital, Sokoto from the North West. Ethical approval was sought and obtained at each center. The three facilities had good obstetric and gynecological services available to women with a record of approximately 15,000 deliveries per year, and of these, over 70% of the women received at least some antenatal care. A sample size of 577 was obtained when an estimated population of 15,000 pregnancies per year from hospital records at 95% confidence level and 4% confidence interval was input into Raosoft ^(R) ^and Epi Info^® ^statistical software packages [35,36]. Allowing for missing data within fields, as well as sub-group analysis, the number was rounded up to 600. This was use as a guide to determine the number of respondents to be targeted within the study period.

### Participants

With the assistance of midwives, pregnant women who were attending the antenatal clinic of selected hospitals were recruited while waiting for ante-natal check-ups. Women attending antenatal clinic between September 2007 and March 2008 at the three centers were eligible for inclusion in the study. Excluded were those who were ill at the time of recruitment or who declined to participate. Verbal informed consents were obtained from women who agreed to participate after the study had been explained to them in English or the local language. In all, the assistance of four interpreters who were hospital midwives were engaged where necessary for about 170 women that could not speak English.

### Data collection

Structured questionnaire was the main instrument used to collect information to evaluate the attitude of respondents towards use of herbal medicine. The instrument which took about ten minutes to complete contained mostly close-ended questions, including "Likert Attitudinal Scale" and straight dichotomous questions. The questionnaires were self-administered by respondents that could read and speak English while the four hospital midwives (to whom the study had been explained) served as interpreters to those who could not speak or write English. The definition of herbal products was explained to the women. Demographic data and responses on the women's knowledge of herbal medicine, use of herbs during pregnancy, reasons for use of herbs, sources of herbs, method of preparation, confidence in efficacy and safety of herb, and knowledge of the effects on the fetus, if any, were obtained. The questionnaire was piloted using a sample of women chosen from the antenatal wards who were not part of the final study. Changes made after pilot test included respondent's opinion about co-administering herbs and conventional western medicines.

Variables having low frequency such as "postgraduate education" was merged with tertiary education, while basic adult literacy education (where respondents were taught to read and write in the local language) was merged with "no formal education". Descriptive statistics and Fisher's exact tests were used at 95% confidence level to evaluate the data obtained. Percent frequencies were also used for analysis of data. Level of significance was set at p < 0.05.

## Results

Five hundred and ninety five questionnaires were completed, giving a response rate of over 99 percent. Most respondents, 399 (66.5%) were in the age range 21-30 years old with about half, 245 (40.8%) having up to secondary level of education. Majority were married 585 (98.3%). The demographic data are given in details in Table 1.

**Table 1:** Demographics of respondents who participated in the study

Demographic variable		Frequency (percentage )
Age (years)	≤ 20	79 (13.2%)
	
	21-30	399 (66.5%)
	
	31-40	111 (18.5%)
	
	41-50	5 (0.83%)

Level of education	No formal Education	101 (16.8%)
	
	Basic adult literacy Education	1 (0.2%)
	
	Primary	65 (10.5%)
	
	Secondary	245 (40.8%)
	
	Tertiary	176 (29.3%)
	
	Postgraduate	5 (0.83%)

Geopolitical zone	North Central	198 (33%)
	
	North West	202 (33.7%)
	
	South Western	195 (32.5%)

Marital Status	Single	5 (0.83%)
	
	Married	585 (98.3%)
	
	Separated	5 (0.83%)

More than two-third of respondents, 405 (67.5%), had used herbal medicines in the crude form that was prepared by respondents or as packaged herbal or dietary/nutritional supplements. One hundred and sixty (26.7%) had never used herbal medicines, while 30 (5.0%) were not sure if they had. For those that had used herbal medicines in recent years, a majority, 151 (37.3%) were from southwest, 145 (35.8%) from north central and 109 (26.9%) were from the northwest zone of the country. Four hundred and eighty two (81.0%), were of the opinion that herbal medicines could be effective, 36 (6.1%) did not belief in the efficacy of herbal remedies, while the remaining 77 (12.9%) were not sure of the efficacy. A high number of the respondents, 337 (56.6%) did not support combining herbal medicines with allopathic drugs in any form, 82 (13.7%) believed it is safe to do so while 177 (30.0%) were not sure. Almost half the respondents, 280 (47.0%), did not belief there could be interaction between herbal remedies and allopathic drugs when concomitantly administered, while 49 (8.2%) believed co administration may result in interaction. One hundred and ninety nine (33.4%) respondents believed herbal medicines possess no side or adverse effects, 181 (30.4%) were of the opinion that adverse/side effects of some herbal medicines could be dangerous, 177 (29.7%) were not sure, and 38 (6.4%) gave no response.

Out of the 405 respondents who had used herbs prior or during the present pregnancy, 73 (18.0%) had experienced some form of untoward effects post administration of herbal medicines. The side effects experienced included vomiting 27 (36.99%), dizziness 17 (23.3%), malaise 10 (13.7%), headache 10 (13.7%), rashes 6 (8.2%) and diarrhea 3 (4.1%).

Three hundred and one respondents (74.3%) were of the opinion that taking herbal preparations in the crude, self-prepared form was preferable, while other respondents believed in using the herbal medicines of choice in form of already packaged herbal products or dietary/nutritional supplements which included products from Forever Living, Golden Neo-Life Drugs (GLND) and Tianshi (92; 22.7%) or produced by local "tradomedical" practitioners (19; 3.2%).

For those who prepared the remedies at home, potable water was the preferred vehicle (272; 45.7%). Non-alcoholic carbonated drinks were preferred by 27 (4.5%), infusions of *Camellia sinenesis *(tea) in water by 25 (4.2%), milk 22 (3.7%), alcoholic beverages 5(0.83%), and lime fruit juice 3 (0.5%) as the vehicles of choice. The herbal medicines were obtained mainly from the local herb sellers in the market by 119 (19.8%) while 100 (16.7%) sourced herbs from the wild and 56 (9.3%) bought their herbs from traditional herbalists. Roadside hawkers accounted for 31 (5.2%), herb shops 27 (4.5%), and pharmacies were 5 (0.83%).

One hundred and seventy four of the total respondents (29.0%) were using herbal medicine at the time of the study and believed that the use of herbal medicines during pregnancy is safe, though with different primary reasons for taking the herbal medicine of choice as stated in Table 2. Majority of those who believed in the use of herbal remedies during pregnancy were 72 (41.4%) and 70 (40.2%) from the Southwest and North central geopolitical zones respectively and 32 (18.4%) from the northwestern zone.

**Table 2:** Reasons given by respondents for use of herbal medicines in pregnancy

Reason for use of herbal medicine	Frequency	Percentage Frequency
More effective than conventional medicine	34	22.4%

Not harmful in pregnancy	32	21.1%

When conventional medicine fails	30	19.7%

Cultural to use herbs	19	12.5%

More accessible treatment than conventional medicine	17	11.2%

Cannot afford conventional medicine	9	5.9%

Complements conventional medicine	8	5.3%

Others	3	2.1%

Note: Figures do not add up to 174 due to 22 missing variables

Marital status, geopolitical zones, and educational qualification of respondents had statistically significant effects on respondents views on whether herbal medicines may possess side effects or not (p < 0.05). Respondents from the North West strongly believed that herbal medicines could have side effects; while those from South West, especially respondents with primary education or less believed that herbal medicine do not possess side effects. The number of respondents from the North central who believed that herbal medicines could have side effects was not significantly different from those who did not. Age of respondents also had significant effect on their opinion on the use of herbal medicines during pregnancy (p = 0.003).

Evaluation of the influence of age, geopolitical zones, marital status and educational qualification of respondents on their views on herbal medicines utilization (Table 3) showed there was statistically significant effect between respondents' geopolitical zones, educational qualifications and their views on the harmful effects of herbal medicines to the fetus (p < 0.05). Respondents from the Northwest, who had secondary education strongly believed that herbal medicines could be harmful to the fetus. Respondents from the Southwest, especially those who had less than secondary level of education believed that herbal remedies could not constitute any danger to the fetus.

**Table 3:** Influence of demographic variables of respondents on views on herbal medicine use during pregnancy

Opinion	Variable	P-value
*Belief in efficacy herbal works*	Age	0.62

	Marital status	0.48

	Geopolitical zones	0.09

	Educational qualification	0.45

*Use in current pregnancy*	Age	0.003*

	Marital status	0.17

	Geopolitical zones	0.02*

	Educational qualification	0.048*

*If herbal medicine can cause harmful effect to the fetus*	Age	0.08

	Marital status	0.21

	Geopolitical zones	0.001*

	Educational qualification	0.002*

*Herbal medicine has side effect*	Age	0.37

	Marital status	0.03*

	Geopolitical zones	0.0001*

	Educational qualification	0.0003*

Note: The views of the respondent as dictated by their demographic variables. The statistically significant effects are in asterix* (significant effect. P value less than 0.05)

## Discussion

This study was limited by two factors, namely that the pregnant women were not grouped according to gestational age and the respondents were not asked to name or identify the particular herbs used. This was considered to be outside the scope of the study which was to determine the extent of use, evaluate factors affecting use in pregnancy and to document the incidence of herb use among this population. Response rate from the study which spread across three out of six geopolitical zones in the country was more than 99 percent.

In developing countries, laws regulating sales and distribution of herbal medicines are poor while access to herbal medicines is largely unrestricted. Indiscriminate use of herbal remedies in different forms is very rampant as confirmed by more than two thirds of the respondents in this study who had used herbal medicines at one time or the other in spite of the fact that side effects and teratogenic potentials of most herbal products are poorly understood. Also, manufacturers of herbal medicines or supplements usually offer a broad range of therapeutic claims which are powerful temptations for consumers who perceive herbal drugs to be better and safer alternatives to conventional drugs prescribed by their physicians. Past studies showed that many patients who had used herbal medicines with good therapeutic outcomes later discovered that the benefits were actually due to the presence of undisclosed orthodox medicines to which the herbal medicines has been adulterated [37,38]. On the other hand, however, patients could also develop renal failure and other untoward effects from use of adulterated products [39-41].

Most respondents in the present study did not believe herbal remedies should be combined with conventional allopathic medicines. This was quite unexpected since a substantial number of respondents believed herbal medicines are safe.

Herbal medicines used by pregnant women were self-prepared mostly by mixing different parts of plants, while some took prepared and prepackaged herbal medicinal products. One of the major reasons cited by pregnant women for taking herbal remedies included higher efficacy of herbal medicines when compared with conventional medicines, safety in pregnancy (because they are natural products), beliefs concerning cultural heritage of herbal medicines and the relatively high cost of allopathic/conventional medicines. This confirmed the findings from previous studies [7,8] where the major reasons cited by patients for using herbal medicines were its cost-effectiveness and perceived absence of side effects.

More than two-thirds of respondents who had used herbal medicines at one time or the other in the present study had confidence in the efficacy of herbal medicines. About forty-three percent of this group was also using herbs at the time of the study, many of whom strongly believed in safety of herbal medicines use in pregnancy. They were however unaware of the teratogenic potentials of herbs. This finding was in support of data from a previous study which indicated that about 10% of pregnant women reported the use of herbal medicinal products in a city in Nigeria [24]. The high prevalence of herbal medicine use in this study may probably be due to the wider coverage, that is, three states as compared with only one city used in the other study by Gharoro and Igbafe [24]. Pregnant women from Northwest believed strongly that herbal remedies have side effects and should be taken with caution during pregnancy while those from Southwest who in their majority supported the use of herbal medicines in pregnancy did not believe so.

Potable water was the preferred liquid by most respondents for the preparation of herbal medicines of choice which is expected since water has been considered an inert liquid that will ensure adequate extraction of water soluble constituents from plant materials. Only few respondents got herbal medicines of choice from chemist/pharmacy shops while the majority obtained their choice herbal remedies from the wild. The low patronage of chemist/pharmacy shops for herbal medicines suggested that physicians do not recommend/prescribe herbal medicines to their clients and most pharmacy outlets also do not sell herbal products in their shops [30]. Conventional healthcare providers, especially pharmacists and physicians, in Nigeria had been found to have poor knowledge of the pharmacology, efficacy, safety profile and drug interaction potentials of most herbal medicines available in the country [34,42]. This might explain why healthcare professionals would not want to recommend or sell such products to their patients. Herbal medications may cause teratogenic effects to the fetus as many conventional allopathic medicines [27,28,43-45]. It is therefore essential for physicians to always ascertain if their patients are taking herbal medicines, and patients who do acknowledge use of herbal drugs must also be understood in terms of their illness, beliefs and rational for taking alternative therapies [7,8,45]. Herbal medicines taken by most respondents were reported to be free of side effects. The experienced side effects included vomiting as the major side effect while dizziness, malaise, rashes, headache and diarrhea were also reported. Some of these side effects might be pregnancy-related.

Due to the pharmacologically active components in herbal medicines, there is possibility of potential harm to the fetus or potential interactions between herbal remedies and conventional pharmaceutical preparations. Caution should therefore be exercised with the use of herbal medicines especially during pregnancy.

## Conclusions

The use of herbal medicines by pregnant women in Nigeria was seen to be quite high. Many patients have confidence in the efficacy of herbal remedies and found them helpful as a cost effective and accessible alternative treatment. Health care providers, especially those that are involved in ante-natal, pre-natal and post partum care should therefore be aware of evidence regarding potential benefits or harm of herbal medicinal agents when used by pregnant women, since many of these herbal remedies are self -prescribed based on the women's own information or belief.

## Competing interests

The authors declare that they have no competing interests.

## Authors' contributions

TOF designed the study, questionnaire and was involved in the writing of the manuscript. RA was involved in the analysis of data and the writing of the manuscript while MEI was involved in the design and distribution of the questionnaire as well as collation of data.

## Pre-publication history

The pre-publication history for this paper can be accessed here:

http://www.biomedcentral.com/1472-6882/9/53/prepub
